# Place of birth and outcomes associated with large volume transfusion: an observational study

**DOI:** 10.1186/s12884-021-04091-y

**Published:** 2021-09-13

**Authors:** Jillian Patterson, Deborah Randall, James Isbister, Michael Peek, Tanya Nippita, Siranda Torvaldsen

**Affiliations:** 1 The University of Sydney Northern Clinical School, Women and Babies Research, St Leonards, New South Wales Australia; 2 Northern Sydney Local Health District, Kolling Institute, St Leonards, New South Wales Australia; 3 Women and Babies Research, c/o University Department of O&G, Level 5, Douglas Building, Royal North Shore Hospital, St Leonards, New South Wales 2065 Australia; 4 The University of Sydney Northern Clinical School, St Leonards, New South Wales Australia; 5 Australian National University Medical School, ANU, Garran, Australian Capital Territory Australia; 6 Department of Obstetrics and Gynaecology, Royal North Shore Hospital, Northern Sydney Local Health District, St Leonards, New South Wales Australia; 7 School of Population Health, UNSW, Sydney, Australia

**Keywords:** Postpartum haemorrhage, Blood transfusion, Tertiary hospital, Maternal morbidity

## Abstract

**Background:**

Guidelines recommend that women at high risk of postpartum haemorrhage deliver at facilities able to handle heavy bleeding. However postpartum haemorrhage is often unexpected. This study aims to compare outcomes and health service use related to transfusion of ≥4 units of red blood cells between women delivering in tertiary and lower level hospitals.

**Methods:**

The study population was women giving birth in public hospitals in New South Wales, Australia, between July 2006 and December 2010. Data were obtained from linked hospital, birth and blood bank databases. The exposure of interest was transfusion of four or more units of red cells during admission for delivery. Outcomes included maternal morbidity, length of stay, neonatal morbidity and need for other blood products or transfer to higher care. Multivariable regression models were developed to predict need of transfusion of ≥4 units of red cells using variables known early in pregnancy and those known by the birth admission.

**Results:**

Data were available for 231,603 births, of which 4309 involved a blood transfusion, with 1011 (0.4%) receiving 4 or more units. Women giving birth in lower level and/or smaller hospitals were more likely to receive ≥4 units of red cells. Women receiving ≥4 units in tertiary settings were more likely to receive other blood products and have longer hospital stays, but morbidity, readmission and hysterectomy rates were similar.

Although 46% of women had no identifiable risk factors early in pregnancy, 20% of transfusions of ≥4 units occurred within this group. By the birth admission 70% of women had at least one risk factor for requiring ≥4 units of red cells.

**Conclusions:**

Overall outcomes for women receiving ≥4 units of red cells were comparable between tertiary and non-tertiary facilities. This is important given the inability of known risk factors to predict many instances of postpartum haemorrhage.

## Background

Postpartum haemorrhage (PPH) remains a leading cause of maternal morbidity [1]. Early identification of risk factors for haemorrhage is an important part of the care of pregnant women and enables preparation for a prompt response to haemorrhage. Key preparation includes ensuring women and clinicians make informed decisions about the most appropriate setting for birth, given the woman’s clinical and obstetric history. Current guidelines recommend that women identified as being at high risk of haemorrhage deliver in centres with an onsite transfusion laboratory and facilities to handle a massive haemorrhage [2–5]. As a result, women with known risk factors for haemorrhage often receive care in tertiary centres. Postpartum haemorrhage however is often unexpected and thus can occur at facilities with varying abilities to handle a haemorrhage, including access to red blood cells and other blood products. Across the spectrum of postpartum haemorrhage, there is also a significant clinical difference between women who require transfusion of a small volume of red cells (1-3 units), compared with those who require large volume transfusions (≥4 Units). Women experiencing PPH at non-tertiary centres may require transfusion of emergency stocks of O rhesus Negative (O RhD Negative) red cells and/or transfer to larger centres. Thus, it would be useful to know the health system impacts of large haemorrhages occurring outside of tertiary centres and the associated maternal and neonatal outcomes. Additionally, few studies have considered the impact of large volume transfusion on health system usage and outcomes.

Obstetric care in New South Wales (NSW), Australia is offered across a network of smaller local and larger regional and tertiary hospitals which vary in the services offered [6], with the potential for long distances between a local and tertiary hospital. An important consideration in this context is the trade-off between the risks of birthing in a small hospital with the potential costs associated with the emergency transport of the woman if required, balanced against removing the woman from her home and local support networks to birth in a larger centre. These considerations are similar to those faced in other countries and across health systems with regionalised care [7]. Researchers have attempted to define a woman’s risk of haemorrhage based -86on demographic and clinical characteristics with limited success [8–12]. Women generally considered to be at ‘high-risk’ of haemorrhage and warranting delivery in larger centres are those with multiple pregnancies, bleeding disorders, placenta praevia, antepartum haemorrhage [13], placental abruption, or preeclampsia/gestational hypertension [14]. These risk factors, however, are uncommon, with many haemorrhages occurring in women without these factors [5, 13, 14]. Other risk factors for haemorrhage include increased maternal age, extremes of parity, induction or augmentation, caesarean section, instrumental delivery, fibroids and previous PPH [14]. These ‘other risk factors’ are more common, but are also more weakly associated with haemorrhage, meaning attempts to predict haemorrhage based on these factors result in high rates of false positives [12]. Previous risk factor models based on routinely collected hospital data commonly lack detail on estimated blood loss and/or volume of red cells transfused [8, 9, 11]. These models cannot distinguish between small and large haemorrhages which require different levels of response and would influence the chosen place of birth. It may also be easier to predict the occurrence of larger haemorrhages, than smaller, less serious ones, but this information has not generally been available in large population-based studies.

This study aims 1) to examine the outcomes and health service use of women receiving a large volume transfusion in response to a serious PPH according to place of birth and 2) to identify risk factors for women at risk of large volume transfusion (≥4 Units) using risk factors available in early pregnancy, and at the birth admission.

## Methods

The study population was all women giving birth in public (government funded) hospitals in New South Wales, Australia, between July 2006 and December 2010. Women were excluded from all analyses if they had a diagnosis of a bleeding or platelet disorder, and those with missing data on key characteristics (maternal age, parity, plurality of birth and history of caesarean section).

Data on maternal and pregnancy characteristics were obtained from the Perinatal Data Collection (‘birth data’), a statutory collection of information on livebirth and stillbirths of at least 20 weeks gestation or 400g birthweight in NSW. Procedure and diagnosis data were obtained from the Admitted Patients Data Collection (‘hospital data’) which collects data on all inpatient admissions to hospitals in NSW, coded according to the International Classification of Diseases version 10 – Australian Modification, and the Australian Classification of Health Interventions. Data on number of units transfused were obtained from the NSW Clinical Excellence Commission BloodWatch Red Cell Utilisation database (‘red cell data’) and the Australian Red Cross Lifeblood (“Lifeblood”) database. Public hospital pathology laboratories submitted data on each red cell unit issued to patients to this database as part of the “BloodWatch” quality improvement program. Not all hospitals submitted data each month, so for the purpose of this study, all births in a hospital are included for each month data were submitted. The Lifeblood database contains additional information on each blood unit distributed to hospitals. Data on hospital admissions from birth to 28 days of life for infants were linked to the maternal record.

The birth, hospital and red cell data were probabilistically linked using personal identifiers by the Centre for Health Record Linkage based on personal identifiers, with de-identified data provided to researchers. Rates of missing and incorrect links were low (<5 per 1000) [15].

The exposure of interest was transfusion of 4 or more units of red blood cells, occurring during the birth admission, and was obtained from the BloodWatch and Lifeblood data. This cutoff was decided in consultation with clinicians, and informed by available data. Data were also available on transfusions given before or after birth where these occurred at other facilities and the patient was transferred. Birth hospital, mode of birth and maternal demographics were obtained from the birth data. Hospitals were classified by location (metropolitan or regional), number of births per year, and obstetric capability (tertiary vs non-tertiary). In NSW hospital capability levels are stratified, with each level encompassing an increased complexity of obstetric care and similarly a greater range of pathology and transfusion services. Levels 1-3, cater for postnatal care and term birth of low-risk women and may not have 24 hour onsite pathology and transfusion facilities (although hold emergency stock of O RhD Negative blood), through to hospitals at levels 4-5 which offer care for low and moderate risk pregnancies, and level 6 tertiary hospitals that provide care for women in all risk categories. In general, women receive care at their closest hospital. A woman was classified as a transfer if the birth occurred outside a tertiary hospital, and the woman was then transferred to another facility. Maternal medical conditions were obtained from the hospital data. Prior PPH and prior uterine surgery were identified using a 5-year lookback period prior to the birth, and uterine fibroids used a 12-month lookback period. Maternal morbidity was measured using a composite indicator which includes procedures and diagnoses, such as shock, acute renal failure, and mechanical ventilation associated with severe morbidity [16]. Although the original indicator includes transfusion of red blood cells or other blood products and procedures to reduce blood flow to the uterus, these were excluded from the indicator for the purposes of this study and reported separately. Readmission was defined as an admission to a hospital facility following discharge home from the birth admission, within 6 weeks of the birth. Adverse neonatal outcomes were measured using a composite measure [17] which includes diagnoses and procedures such as birth trauma, birthweight<1500g, gestational age <32 weeks, sepsis, intraventricular haemorrhage, hypoxic-ischaemic encephalopathy, respiratory distress, necrotising enterocolitis, mechanical ventilation, and major surgery.

Modified Poisson regression with robust error variance was used to identify risk factors for large volume transfusion (vs transfusion of 0-3 units of red blood cells) through two models. The first model adjusted for characteristics that would have been known about the pregnancy at the end of the first trimester (when women most commonly ‘book-in’ to their local hospital), namely maternal age, maternal country of birth, multiple pregnancy, smoking, chronic conditions, morbid obesity, use of assisted reproductive technology, previous PPH, uterine surgery within the past 5 years, a hospital admission with uterine fibroids within 12 months, parity, and socioeconomic status. The second model included additional factors that may have arisen during pregnancy but would be evident when the woman presents for delivery: placenta praevia, pregnancy hypertension, induction of labour, placental abruption and gestational age. For each of these models, the strongest 3 risk factors (based on relative risk) were identified, and women were classified according to whether or not they had one of these 3 risk factors. In addition, women with “no identifiable risk factors” were those who had none of the risk factors recorded, with the exception of maternal age and parity which were considered too general to be useful.

All analysis was performed in SAS 9.4.

## Results

**Fig. 1:**
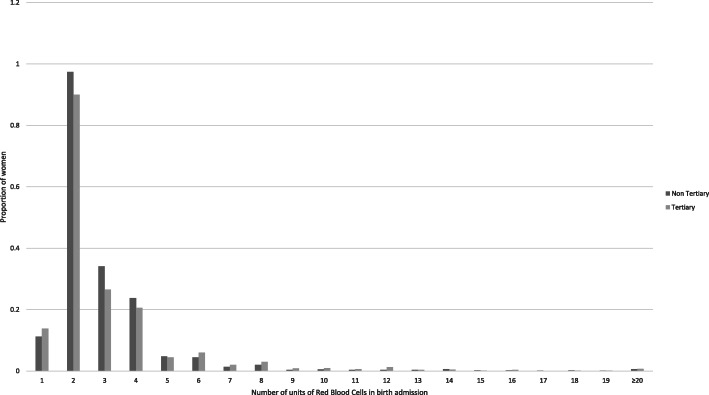
Number of units of Red Blood Cells transfused in birth admission for women receiving a transfusion, according to hospital type

Data were available for 231,603 births between July 2006 and December 2010. Of these, 4309 (1.9%) received a transfusion during their birth admission, with 1011 (0.4%) receiving 4 or more units of red blood cells (Table 1). Women giving birth in hospitals of lower level [1–3] and/or low-medium birth volume (<1000 births per annum) were more likely to receive large volume transfusions, while births in the largest hospitals were less likely to receive large volume transfusion. Overall, 16.4% of births occurred in regional or rural hospitals, however 19.5% of large volume transfusions were given in these settings. Maternal and pregnancy characteristics associated with receiving 4 or more units of red cells included older maternal age, history of uterine surgery, chronic conditions (including cardiac, renal, autoimmune and thyroid disorders, chronic obstructive pulmonary disease), previous postpartum haemorrhage, placental abnormalities in the current pregnancy (placenta praevia or morbidly adherent placenta), use of assisted reproductive technology and gestational hypertension (Table 1).Overall the distribution of number of units transfused in the birth admission was similar between tertiary and non-tertiary facilities (Figure 1).

**Table 1:** Patient and pregnancy characteristics by transfusion volume

		Total N (Col %)	No transfusion N (Col %)	1-3 Units N (Col %)	4 or more units N (Col %)	***p***-value*
	N (Row %)	231603 (100.0)	227294 (98.1)	3298 (1.4)	1011 (0.4)	
Hospital level	1-3	20070 ( 8.7)	19594 ( 8.6)	354 ( 10.7)	122 ( 12.1)	<.001
	4-5	97022 ( 41.9)	95285 ( 41.9)	1372 ( 41.6)	365 ( 36.1)	
	Tertiary	114511 ( 49.4)	112415 ( 49.5)	1572 ( 47.7)	524 ( 51.8)	
	Total non-tertiary	117092 ( 50.6)	114879 ( 50.5)	1726 ( 52.3)	487 ( 48.2)	
Hospital location	Urban	193665 ( 83.6)	190225 ( 83.7)	2626 ( 79.6)	814 ( 80.5)	<.001
	Regional	37938 ( 16.4)	37069 ( 16.3)	672 ( 20.4)	197 ( 19.5)	
Birth volume	<500	7873 ( 3.4)	7572 ( 3.3)	237 ( 7.2)	64 ( 6.3)	<.001
	500-999	13800 ( 6.0)	13527 ( 6.0)	197 ( 6.0)	76 ( 7.5)	
	1000+	209930 ( 90.6)	206195 ( 90.7)	2864 ( 86.8)	871 ( 86.2)	
Maternal age	<20 years	9290 ( 4.0)	9044 ( 4.0)	200 ( 6.1)	46 ( 4.5)	<.001
	20-24	37999 ( 16.4)	37274 ( 16.4)	593 ( 18.0)	132 ( 13.1)	
	25-29	67242 ( 29.0)	66075 ( 29.1)	911 ( 27.6)	256 ( 25.3)	
	30-34	68556 ( 29.6)	67341 ( 29.6)	936 ( 28.4)	279 ( 27.6)	
	35+	48516 ( 20.9)	47560 ( 20.9)	658 ( 20.0)	298 ( 29.5)	
Maternal country of birth	Australia/NZ	147844 ( 63.8)	145071 ( 63.8)	2141 ( 64.9)	632 ( 62.5)	<.001
	NE/SE/South Asia	41967 ( 18.1)	41077 ( 18.1)	673 ( 20.4)	217 ( 21.5)	
	Other	40923 ( 17.7)	40288 ( 17.7)	476 ( 14.4)	159 ( 15.7)	
Smoking during pregnancy		33013 ( 14.3)	32346 ( 14.2)	497 ( 15.1)	170 ( 16.8)	0.03
Previous uterine surgery (5 years)		376 ( 0.2)	359 ( 0.2)	9 ( 0.3)	8 ( 0.8)	<.001
Uterine fibroids (1 year)		313 ( 0.1)	297 ( 0.1)	9 ( 0.3)	7 ( 0.7)	<.001
Pre-existing diabetes		1567 ( 0.7)	1532 ( 0.7)	21 ( 0.6)	14 ( 1.4)	0.02
Chronic hypertension		2121 ( 0.9)	2065 ( 0.9)	42 ( 1.3)	14 ( 1.4)	0.03
Chronic Conditions		3695 ( 1.6)	3517 ( 1.5)	115 ( 3.5)	63 ( 6.2)	<.001
Previous CS		30065 ( 13.0)	29436 ( 13.0)	433 ( 13.1)	196 ( 19.4)	<.001
Parity	Primiparous	98589 ( 42.6)	96394 ( 42.4)	1750 ( 53.1)	445 ( 44.0)	<.001
	1-4 previous births	130740 ( 56.5)	128691 ( 56.6)	1508 ( 45.7)	541 ( 53.5)	
	5+	2274 ( 1.0)	2209 ( 1.0)	40 ( 1.2)	25 ( 2.5)	
Previous PPH		18013 ( 7.8)	16287 ( 7.2)	1283 ( 38.9)	443 ( 43.8)	<.001
Multiple pregnancy		3957 ( 1.7)	3766 ( 1.7)	142 ( 4.3)	49 ( 4.8)	<.001
Assisted Reproductive Technology		3484 ( 1.5)	3365 ( 1.5)	77 ( 2.3)	42 ( 4.2)	<.001
Placenta Praevia		2059 ( 0.9)	1746 ( 0.8)	189 ( 5.7)	124 ( 12.3)	<.001
Morbidly Adherent Placenta		442 ( 0.2)	228 ( 0.1)	109 ( 3.3)	105 ( 10.4)	<.001
Pregnancy Hypertension		16993 ( 7.3)	16384 ( 7.2)	471 ( 14.3)	138 ( 13.6)	<.001
Gestational Diabetes		16641 ( 7.2)	16319 ( 7.2)	241 ( 7.3)	81 ( 8.0)	0.57
Cephalic presentation		221431 ( 95.6)	217373 ( 95.6)	3124 ( 94.7)	934 ( 92.4)	<.001
Induction		60482 ( 26.1)	58998 ( 26.0)	1164 ( 35.3)	320 ( 31.7)	<.001
Pre-labour Caesarean		33755 ( 14.6)	32988 ( 14.5)	511 ( 15.5)	256 ( 25.3)	<.001
Abruption		1029 ( 0.4)	857 ( 0.4)	111 ( 3.4)	61 ( 6.0)	<.001
Gestational age	20-32	5354 ( 2.3)	5084 ( 2.2)	191 ( 5.8)	79 ( 7.8)	<.001
	33-36	12653 ( 5.5)	12257 ( 5.4)	258 ( 7.8)	138 ( 13.6)	
	37+	213555 ( 92.2)	209912 ( 92.4)	2849 ( 86.4)	794 ( 78.5)	
Large for gestational age (>90^th^ centile)		24187 ( 10.4)	23526 ( 10.4)	521 ( 15.8)	140 ( 13.8)	<.001

* chi-square test of difference in number of units transfused across levels of patient characteristics

Women giving birth in tertiary hospitals who experienced large volume transfusion were more likely than those in lower level hospitals to receive transfusion of additional blood products such as platelets and fresh frozen plasma (42% vs 25.9%) and have longer hospital stays; however morbidity, readmission and hysterectomy rates were similar to those receiving ≥4 units of red cells who birthed in a non-tertiary setting (Table 2). Rates of severe morbidity were high in women receiving ≥4 units of red cells regardless of place of birth (26.1% in tertiary and 20.9% in non-tertiary settings). Of women who birthed in a non-tertiary centre 6.6% of those who received ≥4 units of red cells required transfer to a tertiary centre, compared with 4.7% of those who were not transfused and 4.3% of those who received 1-3 units of red cells. Stillbirth rates were similar between those birthing in tertiary and non-tertiary settings, with higher rates amongst those receiving large volume transfusions. Neonatal morbidity was higher amongst tertiary hospital births, with an increase in neonatal morbidity rates with increasing transfusion volumes across both settings.

**Table 2:** Patient Outcomes and health service use by transfusion status

	Tertiary births			Non-Tertiary		
	0	1-3	≥4	0	1-3	≥4
**Births N(%)**	112415 (100.0)	1572 (100.0)	524 (100.0)	114879 (100.0)	1726 (100.0)	487 (100.0)
**Total units transfused (median, IQR)**	0 (0,0)	2 (2,2)	4 (4,6)	0 (0,0)	2 (2,2)	4 (4,6)
**Transfer**^a^**N(%)**				5376 ( 4.7)	75 ( 4.3)	32 ( 6.6)
**Length of stay (median, IQR)**	3 (2,5)	5 (3,6)	6 (5,10)	3 (2,4)	4 (3,6)	5 (4,7)
**Transfusion of other blood products N(%)**	55 ( 0.0)	34 ( 2.2)	222 ( 42.4)	48 ( 0.0)	36 ( 2.1)	126 ( 25.9)
**Severe maternal morbidity**^b^**N(%)**	624 ( 0.6)	83 ( 5.3)	137 ( 26.1)	491 ( 0.4)	78 ( 4.5)	102 ( 20.9)
**Readmission N(%)**	3645 ( 3.2)	99 ( 6.3)	52 ( 9.9)	3605 ( 3.1)	101 ( 5.9)	40 ( 8.2)
**Hysterectomy N(%)**	6 ( 0.0)	. ( .)	33 ( 6.3)	10 ( 0.0)	1 ( 0.1)	28 ( 5.7)
**Stillbirth N(%)**	921 ( 0.8)	35 ( 2.2)	28 ( 5.3)	624 ( 0.5)	31 ( 1.8)	25 ( 5.1)
**Neonatal adverse outcome indicator N (%)**	9672 ( 8.6)	284 ( 18.1)	126 ( 24.0)	4171 ( 3.6)	125 ( 7.2)	58 ( 11.9)

^a^ transfer to other facility only considered amongst non-tertiary births

^b^ Severe maternal morbidity includes shock, acute organ failure, mechanical ventilation and other diagnoses and procedures associated with severe morbidity, but does not include transfusion in this study [16]

Of risk factors known at booking, previous PPH (adjusted relative risk (aRR) 95% CI: 9.7 (8.5,11.0)), maternal chronic conditions (aRR (95%CI) 3.5 (2.7,4.5)), and a diagnosis of uterine fibroids within the past year (aRR (95%CI) 3.1 (1.4,6.9)) were associated with the highest risk of large volume transfusion (Table 3), with 9.4% of women having at least one of these risk factors (Table 4). Among those who received a large volume transfusion, 48.1% had at least one of these risk factors, with a large volume transfusion rate of 2.2% amongst women with one of these risk factors compared with 0.3% in those without. Despite almost half the women (46.1%) having no identifiable risk factors at booking, 20% of large volume transfusions occurred in this group.

**Table 3:** Risk factors for large volume transfusion (≥4 unit transfusion) among 228,884 pregnancies

Risk factor		Booking	Birth
		**RR(95% CI)**	**RR(95% CI)**
**Maternal age**	Under 20	1.3 (0.96,1.77)	1.43 (1.05,1.93)
	20-34	ref	ref
	35+	1.56 (1.35,1.79)	1.38 (1.2,1.59)
**Maternal Country of birth**	Australia/NZ	ref	ref
	Asia	1.35 (1.15,1.59)	1.39 (1.18,1.63)
	Other	0.94 (0.79,1.13)	0.97 (0.81,1.16)
**Multiple pregnancy**		2.21 (1.63,2.98)	1.72 (1.25,2.36)
**Morbid Obesity**		2.13 (1.15,3.93)	1.54 (0.74,3.19)
**Smoking during pregnancy**		1.37 (1.15,1.62)	1.22 (1.02,1.45)
**Chronic conditions**		3.51 (2.72,4.54)	3.15 (2.43,4.09)
**Assisted Reproductive technology**		2.04 (1.47,2.83)	1.61 (1.18,2.2)
**Previous PPH**		9.66 (8.5,10.97)	8.36 (7.33,9.53)
**Uterine surgery (5 years)**		2.24 (1.03,4.87)	2.4 (1.14,5.01)
**Uterine fibroids (1 year)**		3.12 (1.42,6.85)	2.29 (1.06,4.98)
**Parity**	Primiparous	ref	ref
	Multiparous no previous CS^a^	0.65 (0.56,0.75)	0.71 (0.62,0.83)
	Multiparous, previous CS^a^	1.25 (1.05,1.49)	1.29 (1.08,1.54)
**Low socioeconomic status**		1.25 (1.09,1.44)	1.25 (1.08,1.44)
**Placenta Praevia**			9.27 (7.53,11.43)
**Pregnancy hypertension**			1.49 (1.23,1.79)
**Induction**			1.3 (1.13,1.49)
**Placental abruption**			7.01 (5.17,9.49)
**Gestation (weeks)**	20-32		1.65 (1.27,2.15)
	33-36		1.47 (1.2,1.8)
	37+		ref

^a^*CS* Caesarean Section

**Table 4:** Proportion of women with risk factors for transfusion of ≥4 units of red cells at booking and birth (*n* = 231,603)

	Booking	Birth
**% of women with any of the 3 strongest risk factors**	9.4	8.9
**% of large volume transfusions where woman had one of the 3 risk factors**	48.1	56.1
**% of women with no risk factors**	46.1	30.3
**% of large volume transfusions in women with no risk factors**	19.9	9.9

For conditions known at delivery the three strongest risk factors were placenta praevia (aRR (95%CI) 9.3 (7.5,11.4)), previous PPH (aRR (95%CI) 8.4 (7.3,9.5)), and placental abruption (aRR (95%CI) 7.0 (5.2,9.5)), with 8.9% of women having at least one of these conditions, and 56.1% of large volume transfusions occurring in women with at least one of these risk factors. The rate of large volume transfusion amongst women with the strongest risk factors was 2.7% compared with 0.2% amongst those without risk factors. Only 30% of all women had no identifiable risk factor for haemorrhage by the time of giving birth.

Of those women with 1 of the 3 strongest risk factors for haemorrhage (known at booking), 45.7% delivered at a non-tertiary hospital vs 51.1% of those without any of the 3 strongest risk factors. Of those with risk factors for haemorrhage known at delivery 46.7% delivered at a non-tertiary hospital vs 50.9% of those without these risk factors.

## Discussion

Between 2006 and 2010, 1.9% of women delivering babies in NSW public hospitals received a transfusion of at least one unit of red cells during their delivery admission, with 0.4% receiving 4 or more units. Women giving birth in smaller and/or regional facilities were more likely to receive a large volume transfusion, however patient outcomes and health service usage were similar, with the exception of the use of other blood products. Although several risk factors for large volume transfusion were identified, around 10% of women have at least one of these risk factors, limiting their use in predicting transfusion of ≥ 4 Units of red cells.

Internationally, the definition and rates of massive transfusion differ. Around 1 in 250 birthing women (0.4%) in NSW received a large volume transfusion (4 or more units) of red blood cells. Rates of large volume transfusion seen here are similar to the 0.3% of women in the Burgundy region of France experiencing PPH and receiving 4 or more units of blood and/or additional treatment of PPH [18], the 0.59% of women receiving 5 or more units in Scotland (2009-2012) [19], and the 0.2-0.4% receiving 4 or more units in the United States (US) [20]. The United Kingdom’s Obstetric Surveillance System (UKOSS) records women receiving 8 or more units of red blood cells within 24 hours of birth, and identified this in 23 per 100,000 maternities (2012-2013) [21], and data from the Netherlands using the same definition gives an incidence of 65 per 100,000 maternities (2011-2012) [22]. Swedish data using ≥10 units found an incidence of 53 per 100,000 maternities [23]. These differences may reflect different obstetric practice, patient risk profiles and use of alternative strategies to control bleeding.

Risk factors for haemorrhage were identified, however these appeared to have little influence on the place of birth. The strongest risk factors for large volume transfusion which could be identified at the start of pregnancy were previous PPH, uterine fibroids and maternal chronic conditions; while those which could be identified prior to the birth were placenta praevia, previous PPH and placental abruption. Despite the array of risk factors identified being well recognised [1, 13, 14, 24], the presence of these strong risk factors seemingly had little influence on the location of birth, with only slightly fewer women with these risk factors birthing in non-tertiary centres compared with those with the stronger risk factors. This reflects the limited utility of the identified risk factors in decision making regarding place of birth, as there are no risk factors which are both common and strong [12, 25]. Only around 1 in 3 women in our study were without any risk factors for large volume transfusion at their delivery admission, meaning a large number of women could be directed to birth at a higher-level facility, despite their overall risk of haemorrhage being low. Instead, the large number of weak risk factors, and the number of women with no identifiable risk factors who go on to experience haemorrhage highlights the need for all facilities to be prepared to manage massive haemorrhage. This includes prompt identification of haemorrhage, having protocols or guidelines on PPH management including massive transfusion protocols, ready access to blood products, and in the case of smaller facilities, a clear mechanism for transferring women to higher level facilities in a timely manner [2, 3, 20].

Encouragingly, maternal outcomes were similar between women with transfusion managed at a tertiary facility and those managed at a lower level facility. Stillbirth rates were also similar between tertiary and lower level facilities. The higher rates of neonatal morbidity likely reflect the increased complexities of births at tertiary settings. Overall severe maternal morbidity was comparable between tertiary and non-tertiary sites for those receiving 0-3 units of red cells, with a slight increase in maternal morbidity at tertiary centres compared with those at a lower level hospital for those receiving a larger volume transfusion. This is likely due to women known antenatally to be at high risk of bleeding (placenta praevia, abnormally invasive placenta), and hence at higher risk of adverse outcomes, being referred to tertiary centres for management [2–5]. We did not have access to data to assess whether a woman was receiving care at her closest hospital or had been referred in for higher level care. Links between patient outcomes and hospital level of care and/or delivery volume have been varied. A study of PPH and transfusion related morbidity in the United States (US) by hospital delivery volume likewise found the largest facilities had the highest morbidity rates, although this study excluded women at highest risk of bleeding [26], while another US study looking at regionalisation of care found the highest morbidity levels at mid-level facilities (although this study did not specifically focus on transfusion) [7]. A third US study found both the highest and lowest volume hospitals were associated with increased morbidity, as were teaching hospitals [27]. It is also possible that non-tertiary centres transfuse at higher haemoglobin cutoffs (have lower thresholds for transfusion) than tertiary settings. This means some women who received 4 or more units of blood in a non-tertiary setting would not have received as much blood had they been in a tertiary setting. A US study compared transfusion rates by hospital volume and found higher rates in both the smallest and largest hospitals [26], whereas a similar study conducted in the Burgundy region of France found rates of transfusion of 4 or more units increased with increasing hospital level [18]. In our study women at the lowest level and highest level hospitals were at increased risk of transfusion. Higher rates in lower level hospitals may reflect prophylactic transfusion, where a woman receives additional blood once bleeding has stopped in case she needs to be transferred to a large centre (located a significant distance away) [28]. This is consistent with current recommendations on PPH management [2].

Despite similar outcomes, there were differences in the rate of transfusion of 4 or more units and in the use of other blood products (fresh frozen plasma, cryoprecipitate, platelets etc) between tertiary and non-tertiary centres. Women in lower level (or regional) hospitals were also less likely to receive other blood products, which is likely due to accessibility (with smaller facilities less likely to have stock [28]) and lack of expertise. Management of haemorrhage in tertiary settings may also differ, with greater use of point-of-care testing such as ROTEM/TEG (Rotational Thromboelastometry/Thromboelastography) and greater tolerance of anaemia given ready access to blood and haematology specialists. Early intervention with haemostatic factors in higher-level hospitals may have led to haemorrhages being controlled earlier in these settings and not progressing from 1-3 unit to ≥4 unit transfusions. The longer hospital stays seen in tertiary patients may reflect a more serious underlying problem causing the PPH, but similar outcomes suggesting the haemorrhage was well controlled.

This study uses routinely collected data to identify risk factors for large volume transfusion and to understand how place of birth affects maternal outcomes associated with transfusion. The use of unselected hospitals across the whole state and from all levels of service, reflecting the treatment of nearly 230,000 women increases the generalisability of the results. Although routinely collected data commonly lacks volume transfused, the use of the BloodWatch transfusion laboratory data allowed for distinguishing between small and large volume transfusions. Despite these strengths the study has some limitations. To obtain the detail on number of units transfused, we were limited to births up until the end of 2010. While some practices may have changed since this time, the data remain important as changes are made to blood inventory management to reduce wastage due to expiry. Although we were able to account for a woman’s history of PPH, we did not have data on the blood volume lost in the previous birth. We were not able to determine whether a woman delivered at the hospital she was booked to deliver in (to distinguish between low risk women delivering at their local tertiary hospital and those higher risk women referred in) nor whether a massive transfusion protocol was activated. Also, haemoglobin results and use of point-of-care testing and non-blood haemostatic agents were not available.

## Conclusions

Despite possible differences in management between lower level and tertiary hospitals, it is encouraging that overall outcomes for women and their babies following large volume transfusion of 4 or more units of blood are comparable regardless of where the transfusion was initiated. This is particularly important given the inability of known risk factors to predict many instances of PPH. Although women suspected of being at high risk for haemorrhage should be treated at tertiary facilities where possible, it is important that all hospitals are prepared to manage a massive transfusion situation.

## Data Availability

The data that support the findings of this study are available from the NSW Ministry of Health and others, but restrictions apply to the availability of these data, which were used under license for the current study, and so are not publicly available. Data may be requested from the Centre for Health Records Linkage.

## Abbreviations

aRR: Adjusted Relative Risk
NSW: New South Wales
PPH: Postpartum Haemorrhage
US: United States
